# Effects of Dietary Quebracho Tannin on Performance Traits and Parasite Load in an Italian Slow-Growing Chicken (White Livorno Breed)

**DOI:** 10.3390/ani10040684

**Published:** 2020-04-14

**Authors:** Margherita Marzoni, Annelisse Castillo, Alessandro Franzoni, Joana Nery, Riccardo Fortina, Isabella Romboli, Achille Schiavone

**Affiliations:** 1 Dipartimento di Scienze Veterinarie, Università di Pisa, Viale delle Piagge 2, 56124 Pisa, Italy; margherita.marzoni@unipi.it; 2 Dipartimento di Scienze Veterinarie, Università degli Studi di Torino, Largo Paolo Braccini 2, 10095 Grugliasco (TO), Italy; annelisse.castillogarrido@unito.it (A.C.); alessandro.franzoni@unito.it (A.F.); joana.nery@unito.it (J.N.); 3 Dipartimento di Scienze Agrarie, Forestali e Alimentari, Università degli Studi di Torino, Largo Paolo Braccini 2, 10095 Grugliasco (TO), Italy; riccardo.fortina@unito.it; 4 Independent Researcher, Via Cisanello 73, 56124 Pisa, Italy; isabella.romboli@gmail.com

**Keywords:** Quebracho tannin, growing laying hen, pure breed

## Abstract

**Simple Summary:**

Acknowledging that excessive use of chemical synthesis products in all animal fields is damaging for live beings and the environment, there is a need to propose natural elements as substitutes. Quebracho tannin may act against microbes, parasites, viruses and fungi, and has antioxidant and anti-inflammatory properties. There are few studies on dietary use of condensed tannins in poultry. In this study, dietary Quebracho tannin was tested in growing laying hens of a local pure breed reared under free-range conditions. We found that 2% dietary Quebracho tannin is the maximum inclusion level, and beyond this level becomes detrimental for a growing hen’s health. Quebracho tannin reduced fecal outputs of Nematodes and Coccidia, and this attribute could be helpful for the maintenance of a better litter quality by making birds produce highly dry droppings.

**Abstract:**

Tannins have shown numerous biological activities and are very appreciated in food animal production, especially for their antimicrobial, antiparasitic, antioxidant, anti-inflammatory and antivirus effects. The aim of the present study was to evaluate the effect of two levels of dietary Quebracho tannin (QT) on growth and performance traits, and possible effects on intestinal parasite load in Italian White Livorno pullets. A 140-day trial was carried out on 180 35-day-old females, fed on two levels of dietary QT inclusion: 0%, 1% and 2%. Birds were reared under free-range conditions. Dietary Quebracho tannin may be used up to 1% in growing female White Livorno chickens without any adverse effects. The results observed in this study on the use of dietary QT at 2% might have not reflected the real effect on performance traits due to the initial inclusion of dietary QT at 3%. Nevertheless, by reducing QT to 2%, a progressive normalization of body weight gain, feed intake and feed conversion ratio was observed, resulting in compensatory growth. QT was demonstrated to drastically reduce fecal outputs of Nematodes eggs (*Ascaridia* spp. and *Heterakis* spp.) and Coccidia oocytes (*Eimeria* spp.). The inclusion of 2% produced highly dry droppings.

## 1. Introduction

Phytobiotics are bioactive compounds from a plant origin, which may improve bird’s performance when added to the feed [1,2,3]. These substances may originate from all parts of the plant (leaves, roots, tubers, fruits of herbs, etc.) and might be available in various physical states (solid, dried, ground, extracts, etc.) [4]. The active compounds of phytobiotics are mainly secondary plant constituents—terpenoids (mono- and sesquiterpenes, steroids, etc.), phenolics (tannins), glycosides and alkaloids (present as alcohols, aldehydes, ketones, esters, ethers, lactones, etc.) [1,4,5]. The pathways involved in the effect of most phytobiotics are still not fully understood; they may intervene by activation of feed intake and secretion of digestive enzymes [4]; by causing disruption of cellular membrane in pathogen microbes [1]; by decreasing the virulence of microbes through the increase of hydrophobicity of microbial species throughout their cell surface properties [1]; by stimulating growth of favorable bacteria in the gut [1,2,4,6,7]; by acting as immunostimulatory substances [1]; by protecting intestinal tissue from microbial attack [1,2,6]; and by acting as antioxidants [8,9].

Tannins are plant secondary metabolites consisting of part of the plant chemical defense system against pathogens and insects. Tannins have shown numerous biological properties with antimicrobial, antiparasitic, antioxidant, anti-inflammatory and antivirus effects [1]. According to the chemical structure, tannins are a group of phenolic compounds with diverse structures but with the same ability to bind and precipitate proteins. Tannins are mainly classified into three major groups—hydrolysable tannins, condensed tannins or proanthocyanidins, and phlorotannins. The first two groups are found in terrestrial plants, while phlorotannins are found in marine brown algae [8,10]. Hydrolysable tannins are susceptible to hydrolysis by acids, bases or esterases, being easily degraded and absorbed in the digestive tract [11,12]. Condensed tannins are oligomeric or polymeric flavonoids with complex structures and high molecular weights. Contrary to hydrolysable tannins, only strong oxidative and acidic hydrolysis can depolymerize the condensed tannin structures that are also not susceptible to anaerobic enzyme degradation [13]. In the past, tannins have been considered to act as antinutritional compounds in diets for monogastric animals, nevertheless, reports today prove that according to factors such as concentration and type of tannin source, bird’s age, bird’s health and physiological status, beneficial effects in birds might be obtained [8,14,15,16,17,18,19,20]. However, in monogastric animals, especially poultry, few dietary tannin sources have been studied.

The importance of the Italian Livorno breed is associated with the fact that it gave origin to the commonly known hybrid Leghorn, selected for egg production. The Livorno breed presents mean body weights between 1.7–2.0 kg in females and 2.0–2.5 kg in males. Under optimum conditions, annual mean egg production is around 280 eggs, with a mean weight of 55 g. This breed is represented by various plumage colors, and the most common are white, black, silver neck, gold neck and reddish-brown [21]. Livorno birds are commonly characterized for their rusticity, being perfect to be used in free-range systems, taking advantage on two favorable aspects, the first being the productivity of females, and the second being the high-quality meat produced by males.

The aim of the present study was to evaluate the effect of two levels of dietary Quebracho tannin (QT) on performance traits, and since chickens are the usual hosts of Nematodes and Coccidia when reared under free-range conditions, evaluate a potential effect against these parasites in White Livorno pullets. 

## 2. Materials and Methods

### 2.1. Birds and Diets

The experimental protocol (protocol no. 814715) was approved by the Bioethical Committee of the University of Turin (Italy). A 140-day feeding trial was carried out using 180 35-day-old females of White Livorno chicken breed, an Italian local slow-growing breed. Birds were individually identified by a wing tag, weighted and housed in 18 roofed outdoor pens (6 × 3 m, 10 birds/pen) with concreted floor and wood shavings litter. Prior to housing of the birds, pens were washed and sanitized. For the first 34 days of life, all birds were kept together and fed the same commercial diet. At 35 days old, experimental groups were arranged as follow—control group (QT0), which was fed the basal diet (including a starter and growing period; Table 1) and Tannin groups, which were fed the same basal diet of QT0 with the inclusion on top of 1% or 3% of QT. The source of QT was a commercial animal feed product named MGM-S ^®^ (manufactured by Unitan SAICA, Buenos Aires), extracted from the heartwood of *Schinopsis* spp. This product is available as a fine powder with 58% tannins, 20% phlobaphenes, 14% nontannic compounds and 8% water, with an average polymerization degree of 6-7. Fourteen days following the experimental diet containing 3% QT, birds showed no weight gain, therefore the inclusion of dietary QT was decreased to 2%. Experimental groups were defined as group QT1 (1% QT or 1.7% MGM-S powder on top) and QT2 (2% QT or 3.4% MGM-S powder on top). Six replicates were assigned to each dietary treatment. Water and feed were supplied ad libitum. The basal feeds were analyzed in triplicate and mean results presented [22].

### 2.2. Growth Performance and Feed Conversion Ratio

At regular 14-day intervals, from 35 days up to 175 days old, individual body weight and feed consumption per pen were measured to calculate the average daily weight gain (ADWG), the daily feed intake (DFI) and the feed conversion ratio (FCR).

### 2.3. Blood Sampling

At 63 and 175 days old, blood samples from 18 birds per dietary group (3 birds/pen) were collected from *vena cutanea ulnaris* into EDTA Vacutainers. Total blood protein (TBP) was performed according to Salamano et al. (2010) [24].

### 2.4. Excreta Sampling, Dry Matter and Parasite Load

At 70 and 174 days old, excreta samples were collected from 36 pullets per dietary treatment, following the procedure described by Dabbou et al. (2019) [25]. Briefly, birds were kept for 1 h in cages (3 birds of the same pen/cage). Excreta samples, free from debris and foreign substances, were collected from a tray placed under each cage. A total of 12 pools per dietary treatment were collected for each sampling.

Excreta dry matter was determined according to AOAC (2000). Parasite load evaluation was performed once, at the end of the experimental period (at 174 days old). Sample collection followed the same procedure as described above. The McMaster chamber method was used for *Eimeria* spp. oocyst count, evaluated as oocytes per gram (OPG) [26] and *Ascaridia*/*Heterakis* eggs quantification, evaluated as eggs per gram (EPG) [27].

### 2.5. Weight of Major Body Parts and Internal Organs

At the age of 175 days, 12 pullets per dietary treatment were randomly chosen, fasted for 12 h, weighted and slaughtered by electrical stunning followed by bleeding. Birds were mechanically plucked after immersion in hot water. Manual evisceration was performed. Weights of liver and gizzard were recorded and expressed as percentage of live body weight (LBW). Head-neck and feet were removed and separately weighed; the weight was expressed as percentage of LBW. The weight of ready-to-cook carcass (RCC) was recorded and expressed as percentage of LBW. Breast was separated and weighed; the weight was expressed as percentage of RCC. Lengths of small intestine and caeca were measured by extending them on a flat surface; the length was expressed as cm per 100 g of LBW.

### 2.6. Statistical Analysis

Data were analyzed using JMP software 5.0.1. (2002) [28]. Results are presented as mean values ± SEM. The distribution of variables was tested for normality using the Shapiro–Wilk’s test. Then, data obtained were statistically analyzed by one-way ANOVA. Treatments were compared with the control group by Dunnett’s *t*-test (2-way). For all tests, *p* < 0.05 was considered statistically significant. A statistical trend was considered for *p*-values below 0.1.

## 3. Results

### 3.1. Growth Performance and Feed Conversion Ratio

**Figure 1:**
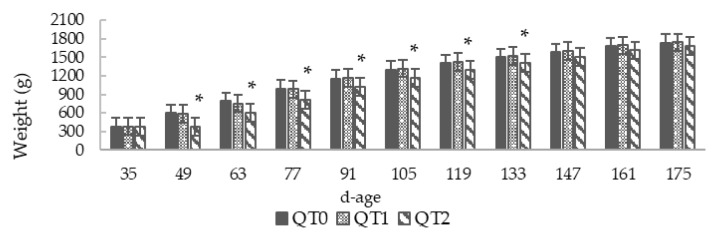
Body weight from birds fed different levels of dietary Quebracho tannin (QT) (mean ± SEM) for a 140-day period starting from 35 days old. * Indicates a difference between treatment and QT0 (Dunnett’s *t*-test, *p* < 0.05). QT0 = basal diet; QT1 and QT2 = the basal diet supplemented with QT at 1% and 2%, respectively.

**Figure 2:**
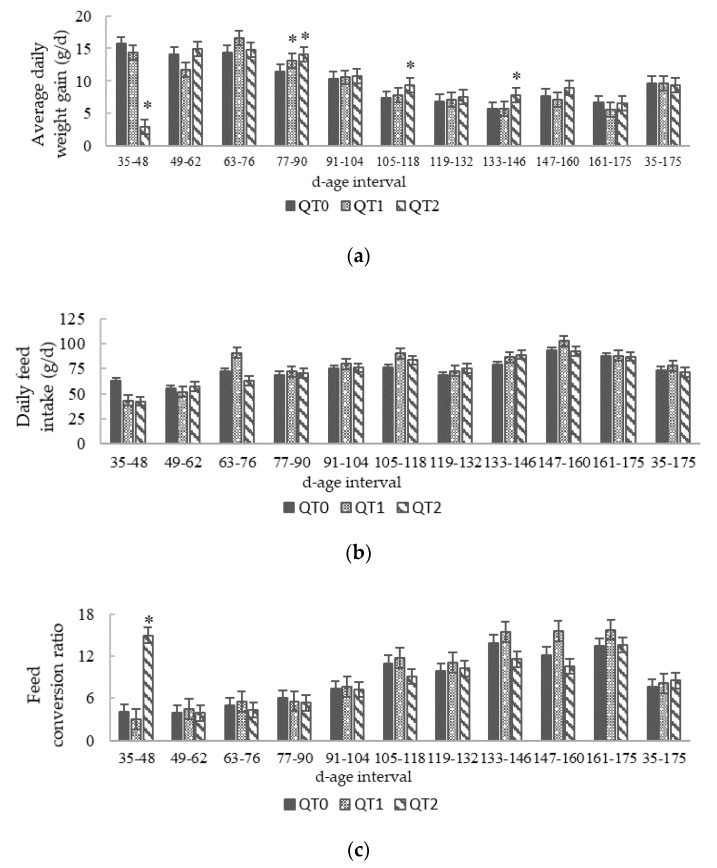
Trends in: (**a**) Average daily weight gain; (**b**) Daily feed intake; (**c**) Feed conversion ratio from birds fed different levels of QT (mean ± SEM) for a 140-day period starting from 35 days old. * Indicates a difference between treatment and QT0 (Dunnett’s *t*-test, *p* < 0.05). QT0 = basal diet; QT1 and QT2 = the basal diet supplemented with QT at 1% and 2%, respectively.

This study lasted 140 days, the birds aged 35–175, and throughout the trial no mortality was recorded. Initially, we planned to feed birds using two levels of dietary QT inclusion, of 1% and 3%. Very quickly, during the initial two weeks, growth performance of birds fed on the diet with the 3% clearly revealed the absence of weight increase (Figure 1 and Figure 2a). Therefore, a QT inclusion reduction from 3% to 2% was applied. Thereafter, the resulting growth pattern remained slightly lower than QT0. This condition lasted up to the age of 147 days, when QT2 reached the weight of the QT0 and no more statistical difference was observed. The LBW at the end of this trial (175 d) was 1728.7 ± 22.8 (QT0), 1732.9 ± 25.7 (QT1) and 1722.7 ± 45.9 g (QT2).

Figure 2a highlights three age intervals (77–90, 105–118 and 133–146) where the ADWG of QT2 group was significantly different compared with the QT0. The inclusion of 1% dietary QT induced no negative effects on growth performance in birds. In fact, the growth pattern of the QT0 and QT1 groups remained almost identical for the whole period, except for the interval 77–90, where QT1 presented a higher ADWG than QT0 (Figure 2a). Considering the whole experimental period, ADWG presented no statistical differences between QT0 and experimental groups; 9.7 ± 0.2 (QT0), 9.7 ± 0.3 (QT1) and 9.4 ± 0.7 g (QT2) (Figure 2a). The mean DFI for all trial periods was 73.5 ± 4.4 (QT0), 77.9 ± 4.1 (QT1) and 71.4 ± 1.1 (QT2) (Figure 2b). The FCR was significantly higher during the first 14 days in the QT2 group (15.0 ± 0.8), thereafter, with the decrease level of QT inclusion from 3% to 2%, no significance was observed (Figure 2c). For all experimental periods, the FCR was 7.6 ± 0.6 (QT0), 8.1 ± 0.6 (QT1) and 8.5 ± 0.6 (QT2). Considering the whole experimental period, 35–175 days old, no dietary effects were observed for ADWG, DFI and FCR between the QT0 and treated groups.

### 3.2. Total Blood Proteins

**Figure 3:**
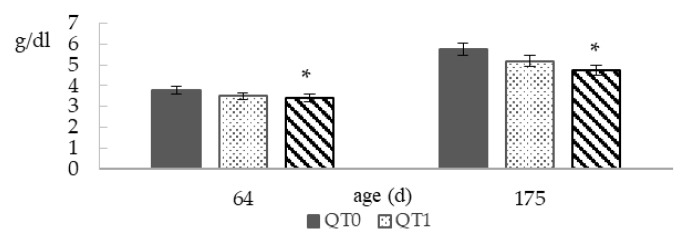
Total blood protein (TBP) from birds fed different levels of QT (mean ± SEM) for a 140-day period starting from 35 days old. * Indicates a difference between treatment and QT0 (Dunnett’s *t*-test, *p* < 0.05). QT0 = basal diet; QT1 and QT2 = the basal diet supplemented with QT at 1% and 2%, respectively.

Figure 3 reports TBP at 64 and 175 days old. The QT2 group showed significantly lower values compared to QT0, at both ages, that is −10.5% and −17.2%, at 64 and 175 days old, respectively.

### 3.3. Excreta Dry Matter Evaluation

**Figure 4:**
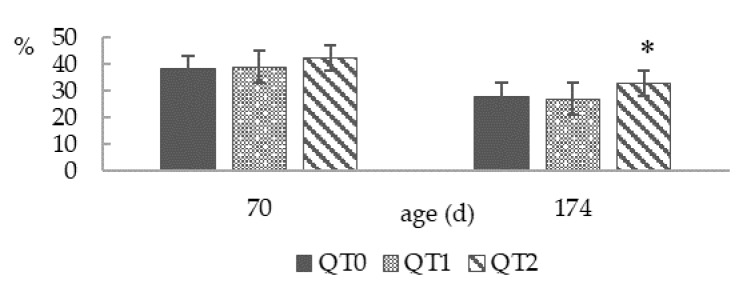
Dropping dry matter at two different ages from birds fed different levels of QT (mean ± SEM). * Indicates a difference between treatment and QT0 (Dunnett’s *t*-test, *p* < 0.05). QT0 = basal diet; QT1 and QT2 = the basal diet supplemented with QT at 1% and 2%, respectively.

Dry matter content of droppings (Figure 4) showed no difference at 70 days old, while at 174 days old, the highest value was displayed by the QT2 group (*p* < 0.01), being 8.3 % higher than QT0.

### 3.4. Excreta Parasite Load

**Figure 5:**
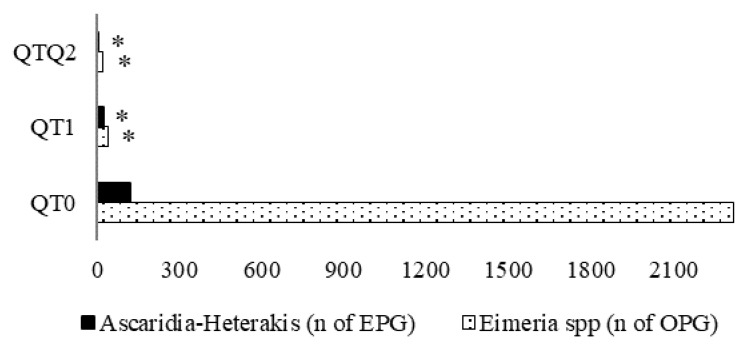
*Ascaridia-Heterakis* (A-H) and *Eimeria* spp. load in fecal samples from 174-day-old birds. * Indicates a difference between treatment and QT0 (Dunnett’s *t*-test, *p* < 0.05). QT0 = basal diet; QT1 and QT2 = the basal diet supplemented with QT at 1% and 2%, respectively. EPG = eggs per gram; OPG = oocytes per gram.

At the end of the experimental period, for both treated groups (QT1 and QT2), EPG and OPG counts were extremely low compared with the QT0. Only 1/6 and 1/59 of EPG and OPG, respectively, were found in QT1 compared with QT0. In QT2 group, no *Ascaridia*-*Heterakis* EPG and very few *Eimeria* spp. OPG were found, less than 1% compared with QT0 (Figure 5).

### 3.5. Weight of Major Body Parts and Internal Organs 

Major body parts and internal organ data (Table 2) revealed no statistical differences (*p* > 0.05) between QT1 and QT2 with QT0. Nevertheless, a statistical trend was observed in the liver weight (*p* = 0.07), with the highest value for QT2. In general, from a macroscopic point of view, organs showed no evident lesions.

## 4. Discussion

According to the little information about the use of QT in poultry feeds [19,20,29], the supplementation levels of dried products and plant extracts in poultry diets varies between 0.1 and 40 g/kg [30]. The 3% QT was part of this range and was found to be acceptable. In our study, the use of 3% dietary QT caused a negative effect on growing performance in birds, suggesting that QT levels above 2% are detrimental for this slow-growing chicken breed. Nevertheless, by decreasing the level to 2%, birds’ performance became acceptable and a progressive increase in LBW was observed. Additionally, after four weeks of consuming the QT2 diet, a compensatory growth was observed in the QT2 group. According to some authors, early feed restriction reduces growth performance, but compensatory growth in the refeeding period accelerates organism growth to reach the standard final live weight of animals [31,32]. During refeeding and compensatory growth, the secretion of insulin is sharply enhanced and plasma growth hormone concentrations remain high. This situation probably allows more nutrients to be used for growth processes [31]. In our study, a similar situation might have happened in QT2 birds for the first two weeks, when they consumed QT at 3%. There was no feed restriction, but birds did not assimilate nutrients, since the ADWG was zero but the DFI was high. Birds dragged this 2-week growth delay until 133 days old, and then they showed equalized LBW compared to that of other groups. Probably, performance of this group could have been better if they had received 2% QT from the beginning instead of the initial 3%. In fact, identification of an adequate level of QT to be used in poultry feeds was one of our targets. Other studies on dietary QT showed no negative effects on growth performances with 2.5% QT in growing Muscovy ducks [20] and with 2% QT in growing pheasants [19]. In grey partridges, 6% QT [29] seemed to alter very slightly when protein food content was relatively high. In fact, no effects were registered in body mass, and the most marked effects were observed in gut morphology. Considering the whole experimental period and the fact that birds were fed on diets supplemented with QT, growth performance is in accordance with data reported by Marzoni et al. (2003) [33] on hens of the same White Livorno Italian local breed. Unlike ruminants, tannins have traditionally been considered antinutritional factors in monogastric nutrition with negative effects on feed intake [34,35]. Nevertheless, the dietary concentrations of tannins that exert a negative effect depend on tannin sources, chemical composition and structure [8]. In captive *Branta canadensis*, QT at 4% induced a decrease in DFI [36]. Conversely, an increase in DFI was reported in male Muscovy ducks fed diets with 2.5% QT [20]. However, in female pheasants the 2% QT did not influence the DFI [19]. Likewise, in this study, excluding the first two weeks with QT 3%, no effects on DFI during the experimental period were observed.

Blood protein in birds represents an important indicator to evaluate health status and production parameters [37,38]. Blood proteins are involved in numerous physiological roles and in homeostasis maintenance, therefore their presence in blood represents an important parameter of evaluation [39]. Normal TBP concentrations in birds ranged between 3.5–5.5 g/DL [40,41]. All groups fall within this range, however, at both sampling times, QT2 showed a significantly lower value compared to QT0 (3.40 vs. 3.80 g/DL and 4.77 vs. 5.77 g/DL at 64 days old and 175 days old, respectively). It is widely known that tannins can render feed constituents less digestible by binding to them, and especially protein absorption tends to be reduced [42,43], and this might have been the cause of the lowest TBP levels in QT2. Another worthy aspect to note is the liver weight, of which alterations can be useful to detect effects of toxic substances [44]. In agreement with Liukkonen-Anttila et al. (2001), no difference was observed in liver weight in QT1 birds. In QT2 birds, a statistical trend (*p* = 0.07) emerged, where liver weight tended to be heavier than QT0. Both the reduced TBP and the trend in increased liver weight suggest an adverse impact in QT2 birds’ hepatic function.

It is worth highlighting the consistency of the birds’ droppings in the QT2 group, which resulted in significantly higher dry matter content, in accordance with results obtained in growing female pheasants [19] and growing male Muscovy ducks [20]. Firmer droppings tend to provide a better litter quality, and consequently, may improve the overall health status and welfare of hens, particularly in intensive production systems [35]. 

Traditionally, the main property attributed to tannins is their ability to contrast parasites, and this capacity has been demonstrated by both in vitro and in vivo studies [45,46,47]. Data obtained in this study agreed completely with former findings. There are substantially two ways with regard to how plant tannins act against helminths, by direct influence on parasite cells [47,48,49] or by improving host resistance against nematodes [50,51]. The capacity of tannins to contrast helminths depends on their chemical composition and structure, parasite species, parasite developmental stage and the host species [49]. In our study, QT capacity against helminths turned out to be very strong, where even the lowest QT inclusion (1%) exerted a good effect. Our results show a strong effect of QT against *Eimeria* spp. Positive results were also reported by Cejas et al. (2011) against *Eimeria* spp. in broilers fed diets with polyphenolic vegetable extract. Naturally occurring phenolic compounds have long been recognized as effective antioxidants [52]. QT is a phenolic compound with antioxidant capability, and this characteristic might be the origin of its anticoccidia capacity. Products with antioxidant properties such as γ-tocopherol and curcumin are effective against *Eimeria maxima* in the mid-small intestinal tract [53]. Additionally, tannins possess anti-inflammatory activities [54,55] that are positively associated with their antioxidant activities [55,56]. For example, it was reported that anti-inflammatory substances like carvacrol, cinnamaldehyde, and capsicum at low doses presented immune-enhancing properties able to protect broiler chickens against coccidiosis challenge infection [57].

Individuals of all species have developed strategies, anatomical characteristics or physiological capacities to extract and utilize feed nutrients. In fact, wild birds have developed larger intestines and caeca, and heavier gizzards due to their diets, which often contain tannins [58,59]. Our results, instead, show evidence of no intestinal length difference between QT groups with the QT0.

## 5. Conclusions

Dietary Quebracho tannin may be used up to 1% in growing female White Livorno chickens without any adverse effects. The results observed in this study on the use of dietary QT at 2% might have not reflected the real effect on performance traits due to the initial inclusion of dietary QT at 3%. Nevertheless, by reducing QT to 2%, a progressive normalization of body weight gain, feed intake and feed conversion ratio was observed, resulting in compensatory growth. Furthermore, an improved dropping dry matter was observed in the QT2 group.

QT demonstrated that it drastically reduced fecal outputs of Nematode eggs (*Ascaridia* spp. and *Heterakis* spp.) and Coccidia oocytes (*Eimeria* spp.), especially with dietary inclusion of QT2. Dietary QT1 in general showed results almost the same as QT0, but exerted an antiparasitic effect that was slightly lower than the QT2. This aspect could be of great interest for poultry farming, mainly in the free-range system, since one of the greatest challenges they deal with is the struggle against parasites.

## Figures and Tables

**Table 1:** Ingredients and chemical composition of basal diets.

Ingredient (%)	0–49 Days	50–175 Days
Corn	52.0	46.0
Full fat soybean	30.5	12.7
Wheat	-	20.00
Soybean meal	9.0	14.0
Alfalfa meal	2.8	2.8
Gluten feed	3.0	2.0
Dicalcium phosphate	1.0	1.0
Sodium bicarbonate	0.5	0.5
Sodium chloride	0.2	0.2
Vitamin-mineral premix ^1^	0.5	0.5
DL-Met	0.35	0.2
L-Lys	0.15	0.1
Chemical composition (% of DM)		
Dry matter	90.89	90.80
Crude protein	22.30	18.05
Ether extract	7.95	4.98
Crude fiber	4.67	4.01
Ash	5.76	5.59
Metabolizable energy ^2^ (MJ/kg)	12.54	12.98

^1^ Supplied per kilogram of diet: Vit. A 11.000 IU; Vit. D3 2.000 IU; Vit. B1 2.5 mg; Vit. B2 4 mg; Vit. B6 1.25 mg; Vit. B12 0.01mg; α-tocopheryl acetate 30 mg; Biotin 0.06 mg; Vit. K2.5 mg; Niacin 15 mg; Folic acid 0.30 mg; Panthotenicacid 10 mg; Choline chloride 600 mg; Mn 60 mg; Fe 50 mg; Zn 1.5 mg; I 0.5 mg; Co 0.5 mg. ^2^ Based on NRC (1994) [23] ingredient composition.

**Table 2:** Major body parts and internal organs evaluation of birds fed different levels of QT from 35 to 175 days old (mean ± SEM).

Item ^2^		Diet ^1^		
		QT0	QT1	QT2
Birds ^3^	n	12	12	12
LBW	g	1729 ± 23	1733 ± 26	1723 ± 46
Breast	% of RCC	22.6 ± 0.52	21.9 ± 0.97	22.3 ± 0.87
RCC	% of LBW	61.5 ± 1.39	62.7 ± 1.57	61.2 ± 0.88
Head-Neck	% of LBW	6.79 ± 0.29	6.95 ± 0.26	6.89 ± 0.16
Feet	% of LBW	2.78 ± 0.08	2.85 ± 0.12	2.82 ± 0.05
Liver	% of LBW	1.52 ± 0.06	1.57 ± 0.07	1.76 ± 0.07
Gizzard	% of LBW	2.25 ± 0.09	2.10 ± 0.13	2.46 ± 0.12
S. Int. L.	cm/100gLBW	6.96 ± 0.26	7.03 ± 0.35	7.65 ± 0.30
Caeca L.	cm/100gLBW	1.92 ± 0.07	2.01 ± 0.07	2.01 ± 0.05

^1^ QT0 = basal diet; QT1 and QT2 = the basal diet supplemented with QT at 1% and 2%, respectively. ^2^ LBW = Live body weight; RCC = Ready-to-cook carcass; S. Int. L = Small intestine length; Caeca L. = Caeca length. ^3^ Each treatment was compared with the QT0 (Dunnett’s *t*-test, *p* < 0.05).
